# Copper and Copper-Based Nanoparticles in Medicine—Perspectives and Challenges

**DOI:** 10.3390/molecules28186687

**Published:** 2023-09-18

**Authors:** Marta J. Woźniak-Budych, Katarzyna Staszak, Maciej Staszak

**Affiliations:** 1 NanoBioMedical Centre, Adam Mickiewicz University in Poznan, Wszechnicy Piastowskiej 3, 61-614 Poznan, Poland; 2 Institute of Technology and Chemical Engineering, Poznan University of Technology, 60-965 Poznan, Poland; katarzyna.staszak@put.poznan.pl (K.S.); maciej.staszak@put.poznan.pl (M.S.)

**Keywords:** copper nanoparticles, drug delivery systems, cancer therapy, antibacterial properties, antiviral properties, perspectives

## Abstract

Nanotechnology has ushered in a new era of medical innovation, offering unique solutions to longstanding healthcare challenges. Among nanomaterials, copper and copper oxide nanoparticles stand out as promising candidates for a multitude of medical applications. This article aims to provide contemporary insights into the perspectives and challenges regarding the use of copper and copper oxide nanoparticles in medicine. It summarises the biomedical potential of copper-based nanoformulations, including the progress of early-stage research, to evaluate and mitigate the potential toxicity of copper nanomaterials. The discussion covers the challenges and prospects of copper-based nanomaterials in the context of their successful clinical translation. The article also addresses safety concerns, emphasizing the need for toxicity assessments of nanomedicines. However, attention is needed to solve the current challenges such as biocompatibility and controlled release. Ongoing research and collaborative efforts to overcome these obstacles are discussed. This analysis aims to provide guidance for the safe and effective integration of copper nanoparticles into clinical practice, thereby advancing their medical applications. This analysis of recent literature has highlighted the multifaceted challenges and prospects associated with copper-based nanomaterials in the context of their translation from the laboratory to the clinic. In particular, biocompatibility remains a formidable hurdle, requiring innovative solutions to ensure the seamless integration into the human body. Additionally, achieving the controlled release of therapeutic agents from copper nanoparticles poses a complex challenge that requires meticulous engineering and precise design.

## 1. Introduction

During the past decade, the development of nanomaterials has expanded into a wide range of clinical applications. To date, the Food and Drug Administration (FDA) and the European Medicines Agency (EMA) have approved approximately 100 nanomedicines, while more than 500 are additionally in clinical trials (Phase I or II) [1,2]. Nanomaterial-based formulations are vital for the worldwide healthcare system [3,4,5]. Advanced nanomedicines can be used as drug vehicles to treat cancer, bacterial and viral infections, and neurological, cardiovascular, immunological, and respiratory system diseases [6,7,8,9,10]. Nanoformulations can be helpful in bioimaging and diagnosis techniques, for example as contrast agents in magnetic resonance imaging (MRI) or positron emission tomography (PET) tracers [11,12,13,14,15,16,17]. The main advantage of nanomedicines lies in their multimodality [18,19]. Nanomaterials simultaneously provide targeted detection and therapeutic capabilities, giving rise to a theranostic approach [20,21,22]. Additionally, nanomaterials can be used to tailor a therapeutic agent to a specific target in a individual patient with a particular disease, contributing to the development of personalised medicine [23,24].

As the field of nanomedicine continues to evolve, the incorporation of copper nanoparticles (NPs) offers exciting new possibilities for enhanced medical treatments and diagnostics. Copper is an essential trace element in all body tissues and is vital in various fundamental biochemical pathways such as glucose, cholesterol, and iron metabolism. It is required for cardiovascular integrity, elasticity of the lung, bone formation, and to work with iron to provide red blood cell synthesis [25]. Both a deficiency and an excess of copper can affect the body’s functions. An excess of copper can cause damage to the liver and various symptoms in the gastrointestinal tract, such as cramps, diarrhoea, vomiting, or abdominal pain. A deficiency can lead to problems with the cardiovascular system, anaemia, tissue damage, and bone defects [26]. Copper use is widespread globally due to its versatility and antimicrobial activity. Copper-based nanomaterials can be applied in agriculture, livestock, water treatment, wood preservation, and textile production. In addition, copper nanomaterials can serve as an alternative to the other noble metal nanomaterials in solar energy conversion, batteries, and electrochemical sensors due to their conductive capacity and lower production costs [27,28]. 

Copper and copper-based nanoparticles have excellent antibacterial activity against *Staphylococcus aureus* (including methicillin-resistant *S. aureus*), *Bacillus subtilis*, *Proteus vulgaris*, and *Escherichia coli* [29,30]. Copper-based nanoparticles were found to have even higher antibacterial properties than Ag NPs [31], and some reports have shown a synergistic antibacterial effect of copper or copper oxide(II) nanoparticles in combinations with silver nanoparticles [32,33].

The widespread use of copper-based nanoparticles in medical applications, including as antimicrobial or antiviral agents, has prompted researchers to expand their studies on the toxicity of copper nanomaterials to microorganisms, animals, and humans. Most scientific research indicates that the toxicity of copper-based nanoparticles is related to the accumulation of nanoparticles around the cell, their dissolution, and their subsequent adhesion to the cell membrane caused by electrostatic interaction. The adhesion of nanoparticles and subsequent release of copper ions disintegrate the cell membrane, facilitating the entry of copper nanoparticles and ions inside the cells. This release of copper ions can cause an increase in the reactive oxygen species (ROS) level, oxidation of proteins, reduced adenosine triphosphate (ATP) production, and DNA damage [34].

The diverse applications of copper nanoparticles increase the likelihood of their leakage into the environment. Once released, nanoparticles undergo various transformations, including dissolution, and can accumulate in plants and animals, causing cytotoxic effects. The toxicity of copper nanomaterials depends on the properties of the nanoparticles (such as size, shape, and surface properties) and the environmental conditions (i.e., composition, temperature, and pH of medium). Woźniak-Budych et al. investigated the stability of copper oxide(I) nanoparticles in various biological fluids, i.e., salivary fluids (pH 6.75), gastric fluid (pH 1.3), intestinal fluids (pH 6.0), and blood plasma (pH 7.2–7.4). They concluded that the stability of Cu_2_O NPs strongly depended on the fluid’s chemistry, i.e., pH, ionic strength, and presence of enzymes [35]. Nanoparticles formed agglomerates in all tested biological media; however, aggregation slowed the dissolution process. The highest dissolution and release of copper ions was detected in gastric and intestinal fluids.

There are four main pathways through which humans are exposed to nanoparticles: inhalation, ingestion, skin contact, and entry through the eyes. However, the respiratory and gastrointestinal tracts are the primary exposure routes. Copper nanoparticles can enter the gastrointestinal tract by ingesting them directly through water and food or inhaling them through the mucociliary clearance of the respiratory tract, which is subsequently ingested after being swallowed [36]. The accumulation of nanoparticles in tissues and organs can cause several serious diseases. For instance, copper nanoparticles could contribute to the progression of neurodegenerative diseases, including Alzheimer’s and Parkinson’s [37].

**Figure 1:**
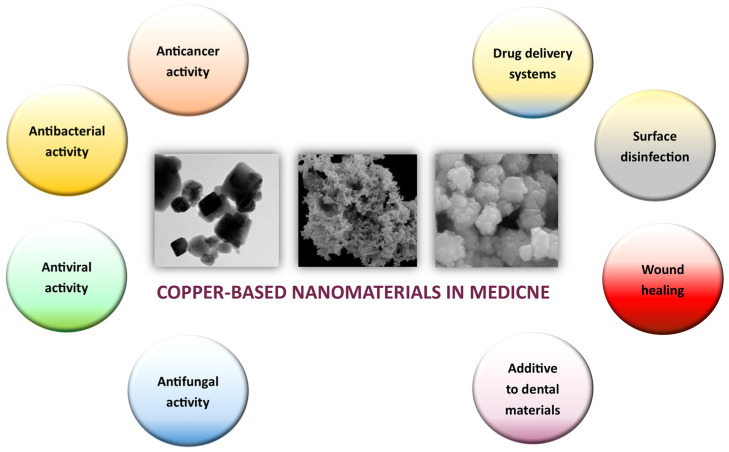
Main properties and applications of copper nanoparticles (Cu NPs) in the field of biomedicine.

This article aims to provide an update on the prospects of copper and copper oxide nanoparticles in medicine, examining three essential properties of copper nanoparticles: anticarcinogenic, antibacterial, and antiviral (Figure 1). We discuss the biomedical potential of copper-based nanoformulations and the challenges associated with successful translation to the clinic. Furthermore, we delve into recent advancements in copper nanotechnology that have the potential to revolutionize medical interventions.

### Materials and Methods

The PubMed, Scopus, and Google Scholar search engines were searched in the bibliometric analysis using the following keywords—combination of copper nanoparticles or copper oxide nanoparticles with: anticancer, antibacterial, antifungal, antiviral, disinfection, antibiotic adsorption, contrast agent, MRI, PET, biomedical, bioimaging, theranostic, personalised medicine, cytotoxicity, biocompatibility, tissue regeneration, wound healing, dental materials, in vivo, in vitro, ex vivo, clinical studies.

The exclusion criteria were manuscripts written in a language other than English, articles not available, and published before 2018 (with a few exceptions of articles essential to present the fundamental studies of copper nanomaterials applications). At the same time, to ensure originality and accuracy, the search focused on research papers while excluding review articles. As a result, items such as articles with titles containing words such as ‘review’, ‘meta-analysis’, or ‘overview’ were not included, with a few exceptions.

**Figure 2:**
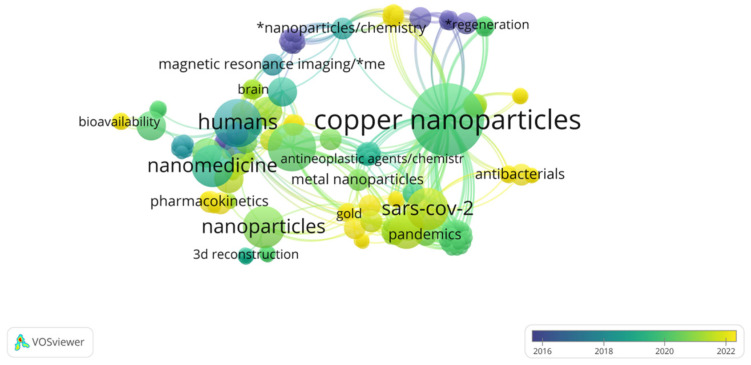
Cluster analysis of semantic web using VOSviewer software (1.6.19).

Figure 2 confirms that the main works mentioned are from 2018. In addition, the exceptions mentioned concerning the previous work are highlighted in purple. The bibliographic analysis indicated strong links between copper nanoparticles and medicine (or rather, nanomedicine), which can supportive the protection of human health and life, pointing to features such as biodegradability, antibacterial properties, or pharmacokinetic studies of how the body interacts with copper NPs for the entire duration of exposure. Chemistry is also important because of the necessity of the correct synthesis of these compounds and determination of the reaction mechanisms occurring in the system under consideration.

Description of the method for conducting a systematic literature review:

Planning

Initial idea formulation

Copper nanoparticles in the biomedical field: challenges, opportunities, perspectives, research directions

PICOC

Population: copper nanomaterials

Intervention: use of copper nanomaterials to improve quality of life and health

Comparison: the development of copper nanomaterials with excellent biocompatibility

Outcomes: in vitro, in vivo, ex vivo, and clinical studies, development of research techniques, medical applications

Context: challenges and opportunities associated with the applications of copper nanomaterials in medicine

Research questions

Which copper-based nanomaterials are used in medicine?

What are the medical applications of copper nanomaterials?

How does the material affect biocompatibility?

What modifications and improvements are proposed?

Can copper-based materials be considered safe?

What is the effect of copper-based materials on animals and humans?

What are the mechanisms of the toxicity of copper and copper nanoparticles?

What are the desired research directions?

What are the challenges associated with the biomedical applications of copper-based nanomaterials?

Digital library sources

Google Scholar, Scopus, PubMed

Inclusion and exclusion criteria

Inclusion criteria—Results obtained by searching using copper nanoparticles or copper oxide nanoparticles with: anticancer, antibacterial, antifungal, antiviral, disinfection, antibiotic adsorption, contrast agent, MRI, PET, biomedical, bioimaging, theranostic, personalised medicine, cytotoxicity, biocompatibility, tissue regeneration, wound healing, dental materials, in vivo, in vitro, ex vivo, clinical studies.

Exclusion criteria—Articles not older than 2018, articles not being a review, with few exceptions: fundamental work on the issues raised.

Quality assessment (QA) checklist

Ensure that:

The search strategy was described correctly. The inclusion and exclusion criteria were appropriate and well-defined. The studies selected for inclusion were relevant to the research question posed.

The included papers were of high quality. They were well-designed and conducted. 

The data were appropriate and well-described. The methods used to include the papers in question were appropriate and well-documented. The consistency of the results was adequately evaluated and presented.

Data extraction form

By application: anticancer, antibacterial, antifungal, antiviral, contrast agent, bioimaging, tissue regeneration, wound healing, dentistry

By material: copper nanoparticles, copper oxide(I) nanoparticles, copper oxide(II) nanoparticles, copper-based core–shell nanostructures, copper salts, copper-functionalised nanomaterials

Conducting

Studies were collected using the Mendeley reference management tool as a database.

Study selection and refinement

The items of literature searched were checked for duplicates and filtered with the exclusion and inclusion criteria described above, leaving a set of articles selected for the research.

## 2. Anticancer Potential of Copper Nanoformulations

**Figure 3:**
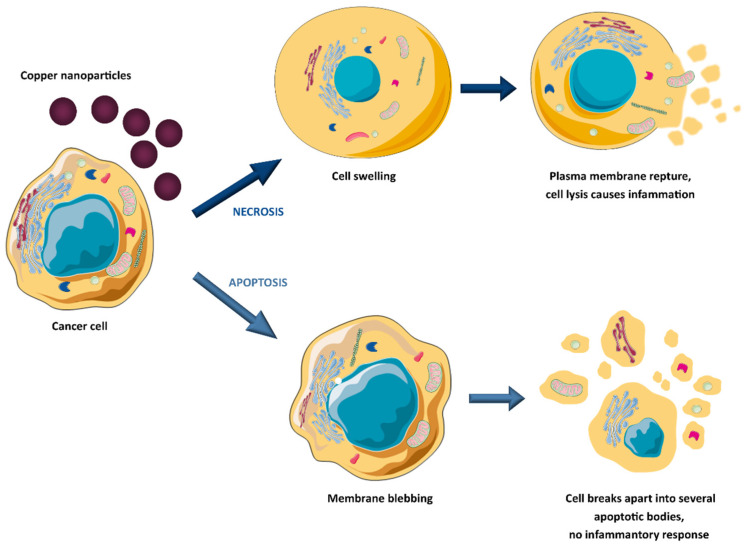
Copper-induced tumour cell death mechanisms.

Copper and copper oxide nanoparticles have drawn much interest in the biomedical fields, offering diverse benefits, including drug stability, proper biodistribution, improved therapeutic index, and active agent delivery to the specific site (active or passive targeting) [35,38,39,40,41]. For instance, copper nanoparticles can be utilised as anticancer therapeutics or effective drug nanocarriers due to their large specific surface area that facilitates conjugation with various biomolecules. Kang et al. [42] designed copper diethyldithiocarbamate nanoparticles (Cu(DDC)_2_ NPs) to overcome resistance in prostate cancer therapy. Based on in vivo studies (xenograft tumour model in male athymic nude mice), it was assumed that Cu(DDC)_2_ NPs prevent cancer cell metastasis and treat resistance by by-passing P-glycoprotein (P-gp) mediated drug efflux transporters, which are responsible for removal of anticancer drugs out of cells. Moreover, it was shown that Cu(DDC)_2_ NPs did not interfere with normal P-gp activities in the body, which is very important because these transporters play a vital role in toxin elimination in healthy tissues. Woźniak-Budych et al. [43,44] demonstrated that copper, copper oxide(I) nanoparticles, and copper–gold core–shell nanostructures exhibit anticancer potential against cervical cancer (HeLa cells, in vitro studies). They can act as therapeutic agents alone or as vehicles for doxorubicin, providing controlled anticancer drug release and inhibiting cancer cell proliferation [44]. The authors also investigated the stability aspect of copper oxide(I) nanoparticles in various physiological fluids, highlighting that glutathione- and hyaluronic acid functionalisation can reduce nanoparticle aggregate formation and corrosion in biological media [35]. The anticancer potential of copper oxide(II) nanoparticles (CuO NPs) against human breast cancer was the topic of studies by the Tabrez group [45]. Treatment with CuO NPs caused a significant decrease in breast cancer cell viability, morphological deformation of cancer cells, enhanced reactive oxygen species (ROS) production, and loss of mitochondrial membrane potential. It indicates that uptake of copper oxide(II) nanoparticles triggers apoptosis. However, copper and copper oxide nanoparticles can also suppress cancer cell viability by necrosis (see Figure 3) [46]. Chen and co-workers designed copper-based nanomaterials (Hc-CuO NPs) consisting of copper oxide(II) nanoparticles (40–45 nm in size) and herbal extract of *Houttuynia cordata* (*Hc*) [47]. The authors showed that Hc-CuO NPs inhibited the proliferation of cervical cancer in vitro by ROS overproduction and the induction of apoptosis by targeting PI3K/Akt (the phosphatidylinositol 3-kinase/protein kinase B) signalling pathways in cancer cells. The cytotoxicity of copper nanocomplexes against cervical cancer resulted from combining CuO NPs and plant extracts with strong antioxidant and anti-inflammatory activities. The synthesis of copper nanoparticles in the presence of natural bioactive compounds has also been investigated by Phull et al. [48] and Abdelhakm et al. [49]. Copper oxide(II) nanoparticles coated with fucoidan derived from *Undaria Pinnatifida* algae exhibited antiproliferative and genotoxic effects on HeLa cells. Additionally, fucoidan-modified CuO NPs demonstrated the ability to modulate the apoptosis of cancer cells via activation of apoptosis-related proteins, including B-cell lymphoma 2 (BCL2), Bcl-2-associated X protein (BAX), cleaved caspase-3, and cleaved poly(ADP-ribose) polymerase (PARP) [48]. In turn, copper nanoparticles functionalised with chrysin as a radiosensitiser improved the effect of radiation on Ehrlich ascites carcinoma in vivo [49]. The combination of Cu NPs and γ-radiation caused a reduction in tumour volume (Ehrlich tumour-bearing mice model) by stimulating apoptosis and inhibiting the nuclear factor-kappa B (NF-κB), p38 mitogen-activated protein kinase (p38 MAPK), and cyclin D1 gene expression. The anticancer potential of CuO NPs was the topic of Benguigui et al.’s research [50]. Copper oxide(II) nanoparticles have been shown to be effective in the treatment of human pancreatic cancer in vitro and in vivo (mice model). The inhibition of tumour growth was related to increased ROS levels and reduction of the mitochondrial potential of the cancer cell membrane. Scientists from China Pharmaceutical University published interesting in vivo research on encapsulated copper sulfide nanoparticles for combined photothermal and chemotherapy [51]. This drug delivery system consisted of copper sulfide nanoparticles, doxorubicin, and a near-infrared dye (NIR) dye (methylene blue analogue, MBA) encapsulated with stearic acid and lauric acid. In addition, to improve the stability of the encapsulated nanoformulation, their surface was covered with a hydrophilic shell made of lecithin and 1,2-distearoyl-sn-glycero-3-phosphoethanolamine–polyethylene glycol 5000 (DSPE-PEG5000). The tumour growth (Ehrlich ascites carcinoma (EAC) tumour-bearing mice model) was significantly inhibited as a result of hyperthermia, triggered by the synergistic action of CuS and MBA (808 nm laser irradiation, 1.0 W/cm^2^) and the release of doxorubicin (DOX) upon thermal-induced decomposition of the outer shell. The tumour inhibition rate reached almost 68% after five days of injection of copper-based nanoformulation. These results indicate that copper sulfide nanoparticles are an effective photothermal agent in advanced combined anticancer therapy. The potential of copper nanoformulations as an anticancer agent has been extensively studied in recent years, with the continuous emergence of innovative approaches. As researchers explore the multifaceted applications of copper and copper oxide nanoparticles in oncology, the landscape of therapeutic possibilities continues to expand dynamically.

## 3. Antibacterial Properties of Copper and Copper-Based Nanomaterials

Copper nanoparticles and copper-functionalised nanomaterials exhibit antibacterial properties against various microbes, including Gram-positive and Gram-negative bacteria and fungi [29,52,53,54]. Zhao et al. [55] investigated the susceptibility of four bacteria (*Staphylococcus aureus*, *Bacillus subtilis*, *Escherichia coli*, and *Pseudomonas aeruginosa*) and four fungi species (*Candida guilliermondii*, *Candida krusei*, *Candida glabrata*, and *Candida albicans*) to copper nanoparticles synthesised in the presence of *Allium Eriophyllum* leaf extract. All tested microbes were sensitive to nanoparticle action. Moreover, copper nanoparticles modified with plant extract exhibited higher antibacterial and antifungal properties than tested antibiotics, such as fluconazole, nystatin, or amphotericin B. The antibacterial properties of copper nanoparticles are generally associated with the nanoparticle’s internalisation within bacterial cells, copper ion release, the generation of oxidative stress by ROS overproduction, and DNA damage [56,57,58]. Similarly, the in vitro inhibition of *E. coli* growth was observed with copper nanoparticles prepared using *Opuntia ficus-Indica* and *Geranium* extracts as a reductor in the nanoparticle synthesis. However, the biocidal properties were observed only at high concentrations of copper nanoparticles, i.e., above 300 μg/mL [59]. It is evident that as the demand for effective antibacterial agents grows in the face of emerging drug-resistant bacteria, copper-based nanomaterials hold great promise for combatting microbial infections.

Zangeneh and co-workers also confirmed the antimicrobial and antifungal activity of copper nanoparticles obtained from the *Falcaria vulgaris* leaf extract [60]. The authors demonstrated the effectiveness of copper nanoparticles in cutaneous wound healing in vivo (rat model). The wound-healing process is complex. It involves blood clot formation, angiogenesis, inflammation, and resurfacing the wound with a new epithelium (re-epithelisation) [61]. Microbes can easily infect the injury and consequently inhibit the healing process. Therefore, it is important to find materials that exhibit antibacterial/antifungal properties and simultaneously stimulate the regeneration of the dermis and epidermis. Based on the in vivo results, the authors concluded that spherical copper nanoparticles with a size of 20 nm provided disinfection of the wound in rats and stimulation of the healing process. Copper nanoparticles modified with *F. vulgaris* increased the number of the fibrocytes and the fibrocyte to fibroblast ratio, thus facilitating wound counteraction. Fibroblast stimulants are responsible for the synthesising fibronectin, hexosamine, and hexuronic acid, which are essential for cell migration and proliferation. In turn, fibrocytes can produce collagen, providing tensile strength in the wound bed [60]. The therapeutic potential of copper nanoparticles in wound healing was also the subject of the Aghdami group [62]. Copper nanoparticles with a size of 80 nm promoted endothelial cell migration and proliferation in vitro. Furthermore, copper nanoparticles accelerated new blood vessel formation (angiogenesis) and stimulated the healing of skin wounds in rat models. Scientists also checked the possible cytotoxicity related to the accumulation of nanoparticles in the rat liver. The levels of alanine aminotransferase (ALT), adenosine triphosphate (ALP), albumin, and total protein in blood samples analysed on 3, 7, 14, and 21 days of the experiment did not change compared with the control groups. It confirms that the topical application of copper nanoparticles is safe and may prevent side effects associated with the accumulation of nanomaterials in tissues and organs [62]. These findings offer promising insights into the development of effective and safe treatments for various skin injuries and infections as researchers explore the many applications of copper nanoparticles in wound healing.

The wound healing potential of plant-derived copper oxide(II) nanoparticles was the subject of Govindasamy and co-authors [63]. The authors evaluated the antibacterial properties of CuO nanoparticles from the giant milkweed plant, commonly known as *Calotropis gigantea*. The plant-derived CuO NPs effectively reduced the growth of *E.coli*, *S. aureus*, *K. pneumoniae*, and methicillin-resistant Staphylococcus aureus (MRSA) bacterial strains. Moreover, the decoration of copper oxide(II) nanoparticles with *Calotropis gigantea* plant extract provided excellent cytocompatibility in vitro [63]. Sen and Sarkar examined the wound healing potential of glutathione and citrate-coated copper/copper oxide(I) nanoparticles in vivo against two multidrug-resistant bacteria, i.e., *Klebsiella* and *Enterobacter* (albino Wistar rat model) [64]. Glutathione and citrate-stabilised Cu/Cu_2_O NPs promoted re-epithelialisation, regeneration of hair follicles and the sebaceous gland, and accelerated wound closure in rats, together with biocidal activity against multidrug-resistant (MDR) pathogens. Copper-based nanohydrogels may offer a new therapeutic approach to accelerate the healing of diabetic wounds. Copper nanoparticles (10 nm in size) were embedded into carboxymethyl chitosan (CMCS)-protocatechualdehyde (PCA) hydrogel matrix to treat skin wounds in diabetic rats [65]. The copper nanoparticle-enriched hydrogel facilitated wound closure by preventing the proliferation of *E. coli* and *S. aureus* and improved angiogenesis. The authors explained that the stimulation of blood vessel formation that occurs during wound healing depends on the copper-mediated activation of the protein-coding gene ATP7A (transporting alpha) by interfering with VEGFR2 (vascular endothelial growth factor receptor 2)-mediated signalling [65].

**Figure 4:**
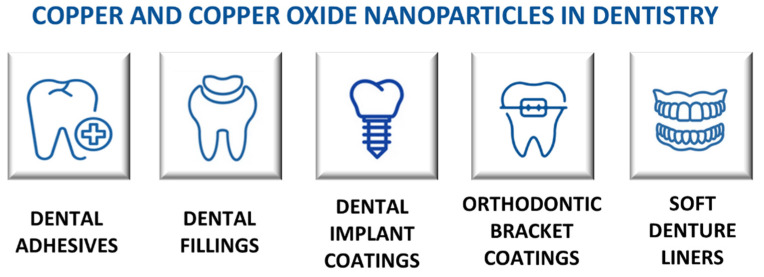
Applications of copper nanomaterials in dentistry.

The antibacterial/antifungal properties and the ability to accelerate the healing of infected wounds of copper nanomaterials have increased their applicability to other areas of medicine, such as dentistry [66]. Copper nanoparticles can be added to dental adhesives, dental filling materials, dental implant coatings, orthodontic bracket coatings, or soft denture liner materials (Figure 4) [67]. Modifying dental materials with copper nanoparticles provides antibacterial properties and enhances (or maintains) their adhesive or mechanical properties. Vidal et al. [68] carried out ex vivo studies with extracted caries-free human third molars to investigate the impact of copper nanoparticles on the final properties of universal dental adhesive. It was shown that dental adhesives containing 0.1% copper nanoparticles exhibited antibacterial activity against *S. mutans* and enhanced adhesive properties. However, the elastic modulus and hardness of the resin–dentin interface remained unchanged, indicating that the mechanical properties were not improved. These ex vivo experiments were performed in a cariogenic oral environment to reflect the natural conditions of the oral cavity. Copper nanoparticles can serve as an antibacterial agent for the disinfection of the root canal system during endodontic therapy [69]. Rojas and co-authors compared the bacteriostatic properties of copper nanoparticles with calcium hydroxide, commonly applied as an intracanal dressing [69]. Antibacterial properties were evaluated on a multispecies biofilm in a root canal ex vivo model. Copper nanoparticles and calcium hydroxide both inhibited bacterial biofilm formation; however, there were no significant differences between their bacteriostatic action against *S. mutans* and *E. faecalis*. The highest decrease in the bacterial population was observed on seven days of exposure. The authors emphasised that copper nanoparticles can be used as an alternative intracanal dressing to suppress dental infections caused by Ca(OH)_2_-resistant multispecies endodontic biofilms [69]. Kang et al. [70] designed microporous titanium dioxide coatings doped with nanostructured copper oxides (I and II) to facilitate osteogenic differentiation and bacteriostatic properties of dental implants. Cu_2_O-doped coatings showed better antibacterial properties against *Staphylococcus aureus* and *Porphyromonas gingivalis* than CuO-doped coatings. Moreover, Cu_2_O-doped TiO_2_ coatings also showed a better effect on bone mesenchymal stem cell proliferation and adhesion than undoped TiO_2_ and CuO-doped coatings. The authors observed the changes in the surface properties of Cu-doped TiO_2_ coatings. The presence of copper reduced their wettability by about 40% [70]. As dental healthcare continues to develop, the incorporation of copper nanoparticles into dental materials will confirm their potential to transform infection control and enhance dental implant functionality.

## 4. Copper Nanomaterials to Combat Viral Infections

**Figure 5:**
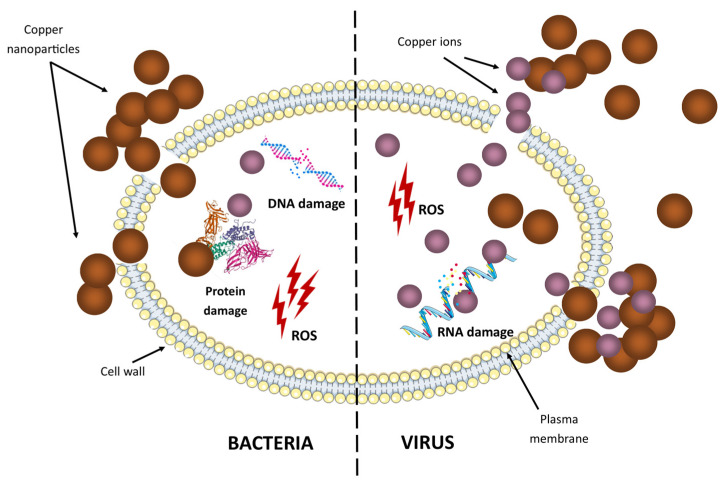
The oligodynamic effect of copper nanoparticles against different microorganisms. The oligodynamic effect is a biocidal effect of metals, such as copper. Copper nanoparticles and copper ions can bind to bacteria and viruses and deactivate pathogens.

The ability of copper nanoparticles to generate ROS makes them a potent antiviral agent (Figure 5). Copper can help treat bronchitis virus, polio, human immunodeficiency virus (HIV-1), and even SARS-CoV-2 [71]. The mechanism is related to stimulating critical immune cells (e.g., T helper cells, B cells, macrophages) to produce antibodies against specific pathogens [72]. Furthermore, the antiviral activity of copper nanoparticles is effective against a wide variety of viruses, such as influenza [73], the herpes simplex virus [74], and hepatitis B [75] and C [34]. Hence, copper nanomaterials offer a promising avenue for the development of broad-spectrum antiviral therapies. This multi-dimensional approach to tackling viral infections highlights the potential of copper nanomaterials in addressing global health challenges.

Merkl and co-workers developed an advanced strategy for nanocoating based on thermophoresis (flame aerosol deposition) to modify the surface of solid and porous materials (e.g., polymer filtration materials, face masks) against the SARS-CoV-2 virus (antiviral materials). A copper oxide(II) nanoparticle thin-film significantly reduced the activity of coronavirus. The virus titer (the concentration of viruses in a sample) decreased by about 50% after 30 min and by 76% over the next 2 h. In turn, Purniawan et al. modified acrylic resin-based paint with copper oxide(I) nanoparticles to cover the stainless steel surface (viricidal materials) [76]. The SARS-CoV-2 virus was completely inactivated after being exposed to the surface coated with copper paint for 1 h. However, before using copper paint in public or hospital settings, further studies are needed to understand the mechanism used to inactivate the virus. SadrHaghighi et al. designed an antiviral hydrophobic facemask filter to prevent the transmission of pathogens that can spread through the respiratory tract [77]. Copper nanoparticles were grafted onto a poly(acrylic acid) melt-blown surface and tested against the SARS-CoV-2 virus. The direct contact of SARS-CoV-2 with the copper-enriched surface caused a significant reduction in virus titer (antiviral materials). In addition, the designed mask filter showed the negligible release of copper ions after 10 wash cycles. This indicates the strong adhesion of copper nanoparticles to the polymer surface [77]. Toledo et al. have performed similar studies [78]. The authors embedded Cu and CuO nanoparticles into poly(methyl methacrylate) (PMMA) and epoxy-based polymer matrix and checked their antiviral potential against HCoV-OC43 human coronavirus (in vitro studies, antiviral properties). Copper-enriched polymers were shown to effectively minimise the long-term activity of immobilised coronavirus. Other studies have indicated the biocidal properties of copper-impregnated fibres against human immunodeficiency virus type 1 (HIV-1) in culture medium [79]. The antiviral mechanism may be related to the damage of the HIV virions by the destruction of their envelope and the denaturation of the viral nucleic acids. It is evident that researchers continue to explore these innovative applications of copper nanomaterials in combating viral infections, a deeper understanding of their mechanisms and potential clinical implications emerges.

The toxic activity of copper nanoparticles against viruses has been studied by Japanese researchers [80]. They investigated the antiviral properties of copper iodide nanoparticles against feline calicivirus virus (FCV), which causes respiratory infections and oral disease in cats. It was shown that CuI nanoparticles at a concentration of 1 mg/mL decreased the infectivity of FCV in cells (Crandell–Rees feline kidney cells, in vitro studies). This effect was associated with the release of Cu^+^ ions, with the subsequent generation of ROS and viral capsid protein oxidation. The authors highlighted the significant role of Cu^+^ ions in toxicity against the FCV virus [80]. Copper nanoparticles can also be effective in the inactivation of the H1N1 influenza virus. Ha et al. investigated the viability of H1N1-infected bronchial and kidney cells treated with spherical copper nanoparticles of around 100 nm in size [81]. The infected cells revealed a significantly decreased level of influenza virus-specific nucleoproteins after 30 min of exposure. This confirms the applicability of copper nanoparticles in the prevention of viral-associated infectious diseases [81]. These studies continue to expand our knowledge of the potential of copper nanoparticles to combat a wide range of viral infections, and the prospects for using copper-based antiviral strategies in clinical settings are becoming increasingly promising.

## 5. Challenges and Future Directions

Despite reports stating that copper and copper-based nanomaterials exhibit excellent therapeutic potential, scientific studies are limited to the in vitro, in vivo, or ex vivo stages. The clinical translation of copper-based nanoformulations is still challenging. Much more effort should be focused on in vivo and ex vivo studies for the detailed investigation of their pharmacokinetics, pharmacodynamics, and long-term toxicity. Compared with other metallic nanoparticles, such as gold, silver, or platinum, copper nanoparticles and copper-based nanomaterials have not been extensively studied. The number of scientific reports studying the biomedical applications of copper nanomaterials is growing; however, there is still a long way to go before they can be utilised in clinical trials. Toxicity studies about copper nanoparticles are fragmentary. Only a few studies illustrate the long-term toxicity of copper nanoparticles, the formation of a protein corona on their surface in contact with physiological fluids, their accumulation in tissues and organs, or detailed studies of the release of copper ions, considering their degree of oxidation (Cu^+^ or Cu^2+^). There is also a lack of literature on the antibacterial and antiviral mechanisms of action of copper nanoparticles of different phase compositions, morphologies, and sizes. All of these parameters affect the biomedical potential of nanoparticles. For instance, the phase composition of copper nanoparticles influences their susceptibility to oxidation after the release of specific copper ions (Cu^2+^ or Cu^+^), which affects their antibacterial and antiviral potential. The morphology and surface properties also play a crucial role. Hydrophobic, porous copper nanoparticles are more prone to bacterial biofilm formation than hydrophilic nanomaterials. However, porosity may facilitate the modification of their surface with bioactive molecules. 

One critical avenue for future exploration involves an increased focus on in vivo and ex vivo studies, which will enable a thorough investigation of copper nanoparticle pharmacokinetics, pharmacodynamics, and long-term toxicity profiles. Copper nanoparticles have not received as much attention in biomedical research compared to other metallic nanoparticles such as gold, silver, or platinum. Although there is a steady expansion of the body of scientific literature concerning the biomedical use of copper nanomaterials, it is necessary to overcome significant challenges before these materials can be advanced to clinical trials.

To enhance our understanding of the long-term toxicity of copper nanoparticles, future research should prioritize the formation of protein coronas after interacting with physiological fluids, as well as investigating tissue and organ accumulation patterns, and the release of copper ions (accounting for the degree of oxidation, whether Cu^+^ or Cu^2+^). The focus should be on achieving more detailed results in these areas. Furthermore, it is crucial to comprehend the antibacterial and antiviral mechanisms of action of different copper nanoparticles, given their unique phase compositions, morphologies, and sizes. These factors play an essential role in determining the biomedical potential of copper nanoparticles, making further research in this direction imperative for maximizing their therapeutic benefits.

The morphology and surface characteristics of copper nanoparticles deserve attention. Hydrophobic and porous variations may be vulnerable to bacterial biofilm formation; therefore, innovative strategies are required to address this issue while preserving their bioactivity. Furthermore, porosity can be used to facilitate surface modifications with bioactive compounds, thus increasing their versatility in medical applications.

Another critical aspect limiting the clinical trial phase of copper nanomaterials is the public perception of copper nanoparticles as highly toxic. Most people are willing to use disinfectants enriched with silver nanoparticles, even though their application should be restricted due to potential biosafety issues. In contrast, copper is an essential trace element for the human body and is important to human health [25]. The scientific community is also more interested in using well-known gold or silver nanoparticles in their research than copper. The number of scientific reports using gold and silver nanoparticles in biomedical applications considerably exceeds the number of works related to copper nanoparticles.

## 6. Conclusions

To address these challenges, it is important for the scientific community to recognize the potential of copper nanoparticles and actively explore their unique properties for biomedical applications. Although gold and silver nanoparticles have constituted the majority of studies, expanding research into the multiple applications of copper nanoparticles will be critical to realizing their full potential in advancing medical science and improving healthcare outcomes.

This article aimed to present the great potential of copper and copper-based nanomaterials in various biomedical applications. Further exploration of the topic at the in vivo and ex vivo stages may elucidate the new unique features of copper nanomaterials and challenges associated with copper nanotherapy, providing general clues to successful clinical translation.

## Data Availability

No new data were created.
